# Scaling Up Molecular Diagnostic Tests for Drug-Resistant Tuberculosis in Uzbekistan from 2012–2019: Are We on the Right Track?

**DOI:** 10.3390/ijerph18094685

**Published:** 2021-04-28

**Authors:** Sharofiddin Yuldashev, Nargiza Parpieva, Salikhdjan Alimov, Laziz Turaev, Khasan Safaev, Kostyantyn Dumchev, Jamshid Gadoev, Oleksandr Korotych, Anthony D. Harries

**Affiliations:** 1 Republican Specialized Scientific Practical Medical Centre of Phthisiology and Pulmonology under Ministry of Health of the Republic of Uzbekistan, 1 Majlisiy str, Tashkent 100086, Uzbekistan; nargizaparpieva@gmail.com (N.P.); s.alimov.med@mail.ru (S.A.); laziz.turaev@gmail.com (L.T.); k_safaev@mail.ru (K.S.); 2 The Charitable Organization “Ukrainian Institute of Public Health Policy”, Biloruska St, 5, 02000 Kyiv, Ukraine; dumchev@uiphp.org.ua; 3 World Health Organization Country Office to Uzbekistan, M. Tarobiy St, 16, Tashkent 100100, Uzbekistan; gadoevj@who.int; 4 World Health Organization Regional Office for Europe, UN City, Marmorvej 51, DK-2100 Copenhagen, Denmark; korotycho@who.int; 5 International Union against Tuberculosis and Lung Disease, 68 Boulevard Saint Michel, 75006 Paris, France; adharries@theunion.org; 6 London School of Hygiene and Tropical Medicine, Keppel Street, London WC1E 7HT, UK

**Keywords:** Uzbekistan, central Asia, Xpert MTB/RIF, line probe assays, molecular diagnostic tests, nucleic acid amplification tests, multidrug-resistant/rifampicin-resistant TB (MDR/RR-TB), extensively drug-resistant TB (XDR-TB), operational research, SORT IT

## Abstract

Uzbekistan has a large burden of drug-resistant tuberculosis (TB). To deal with this public health threat, the National TB Program introduced rapid molecular diagnostic tests such as Xpert MTB/RIF (Xpert) and line probe assays (LPAs) for first-line and second-line drugs. We documented the scale-up of Xpert and LPAs from 2012–2019 and assessed whether this led to an increase in patients with laboratory-confirmed multidrug-resistant/rifampicin-resistant TB (MDR/RR-TB) and extensively drug-resistant TB (XDR-TB). This was a descriptive study using secondary program data. The numbers of GeneXpert instruments cumulatively increased from six to sixty-seven, resulting in annual assays increasing from 5574 to 107,330. A broader use of the technology resulted in a lower proportion of tests detecting *Mycobacterium tuberculosis* with half of the positive results showing rifampicin resistance. LPA instruments cumulatively increased from two to thirteen; the annual first-line assays for MDR-TB increased from 2582 to 6607 while second-line assays increased from 1435 in 2016 to 6815 in 2019 with about one quarter to one third of diagnosed patients showing second-line drug resistance. Patient numbers with laboratory-confirmed MDR-TB remained stable (from 1728 to 2060) but there was a large increase in patients with laboratory-confirmed XDR-TB (from 31 to 696). Programmatic implications and ways forward are discussed.

## 1. Introduction

Despite being an ancient disease, tuberculosis (TB) remains a huge global public health threat. Pulmonary tuberculosis (PTB) accounts for the majority of TB disease in adults and children and is the principal source of ongoing transmission of the infection. A prompt and accurate diagnosis and rapid linkage of diagnosed patients to effective treatment is therefore crucial to any successful TB control strategy. 

Sputum smear microscopy has been the global mainstay of PTB diagnosis for over 100 years due to its availability and simplicity. Unfortunately, it has several shortcomings that include a low sensitivity and an inability to distinguish between drug-susceptible and drug-resistant strains of *Mycobacterium tuberculosis* (*MTB*). With the large and growing global problem of multidrug-resistant TB (MDR-TB, TB resistant to rifampicin and isoniazid) and extensively drug-resistant TB (XDR-TB, this is MDR-TB with an added resistance to fluoroquinolones and second-line injectable agents) [1], the World Health Organization (WHO) has recommended that TB programs replace smear microscopy as the initial diagnostic test with approved rapid molecular diagnostics that allow for a simultaneous diagnosis of *MTB* and drug resistance [2]. 

A “game changer” in the diagnosis of TB has been the introduction, deployment and scale-up of the Xpert MTB/RIF assay (Cepheid Inc., Sunnyvale, CA, USA), a fully automated, commercial nucleic acid amplification test that allows the simultaneous diagnosis of *MTB* and detection of rifampicin resistance within two hours [3]. Using a culture as the reference standard, it has a high sensitivity and specificity and the assay was recommended by the WHO from 2013 as the initial diagnostic test for all people needing investigation for TB [4]. Rifampicin-resistant TB (RR-TB) as detected by Xpert MTB/RIF assays is regarded as a proxy for MDR-TB and patients with RR-TB are treated with MDR-TB treatment regimens. The treatment for drug-susceptible TB is with a six-month regimen of rifampicin and isoniazid supplemented with pyrazinamide and ethambutol for the first two months. MDR-TB and XDR-TB treatment includes both short and long treatment regimens with second-line drugs such as kanamycin, levofloxacin, clofazamine, bedaquiline, cycloserine and prothionamide. 

The standard Xpert MTB/RIF assay, however, cannot diagnose other forms of drug resistance. For this, mycobacterial culture and phenotypic drug susceptibility testing (CDST) using solid or liquid culture media has traditionally been used. However, the major disadvantage of this method is that CDST results can take several weeks during which time patients may be lost to follow-up and continue to transmit infection within the family and the community. Line probe assays (LPAs) (Hain LifeScience GmbH, Nehren, Germany) are an alternative and much faster molecular diagnostic method taking between two to three days for results in good functioning and well-resourced laboratories [5]. LPAs use nucleic acid amplification and gene mutation detection and are recommended by the WHO as the initial diagnostic test for detecting drug resistance to rifampicin and isoniazid [6] and for detecting an additional resistance to second-line drugs if a prior diagnosis of rifampicin-resistant TB or MDR-TB is made [7]. 

Uzbekistan, in central Asia, has seen a significant decline in the number of reported TB patients over the last 16 years from 94 per 100,000 people in 2003 to 49 per 100,000 in 2019, respectively [1]. In spite of this progress, the country has a large problem with drug resistance with a prevalence of MDR-TB of 23% (range 18–29%) in new patients and 62% (range 52–71%) in previously treated patients reported from a drug resistance survey in 2010–2011 [8]. The country is regarded as one of thirty countries in the world with the highest MDR-TB burden [9]. To keep abreast of this public health threat, the National TB Program (NTP) has introduced and deployed new molecular diagnostic tests (Xpert MTB/RIF and LPAs) in the laboratories to complement sputum smear microscopy and phenotypic CDST. Xpert MTB/RIF was first introduced in 2012 followed by LPAs for first-line drugs in 2012 and LPAs for second-line drugs in 2016. There has never been any published systematic documentation of the scale-up, use and characteristics of molecular diagnostic TB tests in Uzbekistan and this is the information that the NTP needs to review as part of its response to the challenge of drug-resistant TB. 

The aim of the study therefore was to document the scale-up, use and outcomes of Xpert MTB/RIF and LPAs in Uzbekistan between 2012 and 2019 and to assess whether this was accompanied by an increase in patients with laboratory-confirmed MDR/RR-TB and XDR-TB and numbers enrolled on MDR-TB and XDR-TB treatment. Specific objectives were to determine between 2012 and 2019: (1) the numbers of GeneXpert and LPA instruments deployed annually and regionally in the country; (2) the numbers of molecular diagnostic tests done annually using Xpert MTB/RIF and LPAs; (3) the results of Xpert MTB/RIF and LPAs in terms of annual tests done, confirmed diagnoses of *MTB* and findings from drug susceptibility testing; (4) the numbers and proportions of patients diagnosed annually with laboratory-confirmed MDR/RR-TB and XDR-TB and numbers enrolled on MDR-TB and XDR-TB treatment. 

## 2. Materials and Methods

### 2.1. Study Design

This was a descriptive study using secondary aggregate program data taken from the electronic database at the National Reference Laboratory and paper-based laboratory record forms.

### 2.2. Setting

#### 2.2.1. General Setting

Uzbekistan is situated in central Asia with an estimated population of 34 million as of January 2020. The country is administratively divided into 12 regions, the city of Tashkent and the Republic of Karakalpakstan. In the past two decades, the Uzbek health system has undergone major reforms, encompassing all levels of health care as well as governance and financing. To sustain major improvements in the quality of care, the Uzbek health system makes ongoing efforts to update treatment protocols, revise medical education, ensure continuous professional development and undertake quality assurance and improvement frameworks [10].

#### 2.2.2. TB Control

There is an NTP chaired by the Republican Specialized Scientific Practical Medical Centre of Phthisiology and Pulmonology under the Ministry of Health of the Republic of Uzbekistan. The NTP encompasses a network of regional centers of phthisiology and pulmonology, city TB dispensaries and district TB departments. Within these facilities, there are fifteen MDR-TB and six XDR-TB wards. TB patients receive standardized treatment in accordance with national guidelines that are based on established and updated WHO Guidelines [11,12,13,14,15,16]. TB diagnosis and treatment is free of charge for all patients. Currently, the country reporting system does not allow routine drug resistance surveillance. According to the most recent Global TB Report, 12% of new patients and 22% of previously treated patients had MDR/RR-TB [1]. 

#### 2.2.3. Laboratory Diagnosis

The TB laboratory network consists of the National Reference Laboratory at the NTP and the Reference Laboratory in Nukus in the Republic of Karakalpakstan. There are five regional laboratories in the Surkhondaryo, Bukhara, Fergana, Samarqand and Tashkent regions and many district and sub-district level health facilities with sputum smear microscopy and/or Xpert MTB/RIF services. The number of GeneXpert instruments has increased over the years since the first deployment in 2012 to regional and district laboratories while LPAs have been deployed in reference and regional laboratories.

**Figure 1:**
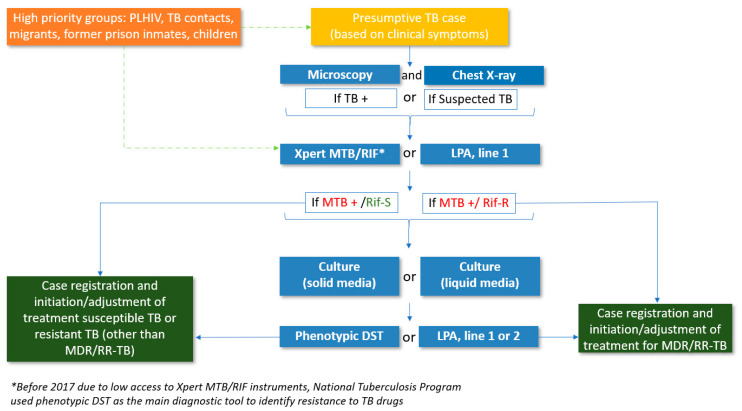
Diagnostic algorithm of people with presumptive TB, Uzbekistan, 2018. TB = tuberculosis, PLHIV = people living with HIV, TB + = smear-positive TB, LPA = line probe assay, *MTB* = *mycobacterium tuberculosis*, Rif-S = rifampicin-susceptible, Rif-R = rifampicin-resistant, DST = drug susceptibility testing, MDR/RR-TB = multidrug-resistant/rifampicin-resistant TB.

The diagnostic algorithm for presumptive TB patients using smear microscopy, Xpert MTB/RIF, LPAs and CDST is shown in Figure 1. 

In brief, presumptive TB patients are screened clinically by chest radiography and by sputum smear microscopy at the primary health care level and are provided with a non-specific treatment. Those who are smear-positive or have an abnormal chest X-ray suggestive of TB or who continue to have symptoms despite an initial course of non-TB antibiotics are referred to a TB doctor who requests an examination by Xpert MTB/RIF or LPA. Over the last few years, however, Xpert MTB/RIF has become the first test of choice for presumptive TB patients; this started in 2012 and by 2016, Xpert MTB/RIF had become the first test of choice in all regions of the country. Patients with rifampicin sensitive TB may be further tested for resistance to other first-line drugs using an LPA and CDST. Patients with rifampicin-resistant TB are further tested for resistance to second-line drugs with an LPA and CDST. For an Xpert MTB/RIF assay, sputum specimens are taken directly from the patient and after preparation are placed in the Xpert cartridges. For the LPA assays, in general, the specimens are either sputum that has been diagnosed as smear-positive for acid-fast bacilli or positive cultures of *MTB* obtained from standard CDST procedures [5]. The information on drug resistance among notified new and previously treated TB patients is recorded in the National Reference Laboratory register and TB case register. 

### 2.3. Study Population

For objectives 1–3 (the numbers of molecular diagnostic instruments and tests done), the study population consisted of the numbers of GeneXpert and LPA instruments and the numbers of diagnostic tests performed annually and for objective 4 (the numbers of patients with laboratory-confirmed drug-resistant TB and numbers enrolled on treatment), the study population consisted of the numbers of patients with laboratory-confirmed MDR/RR-TB and XDR-TB and the numbers enrolled on MDR-TB and XDR-TB treatment in Uzbekistan between 2012 and 2019. 

### 2.4. Data Variables, Data Collection and Sources of Data

Data variables are shown in relation to the objectives. For the first three objectives, data variables included the year, region, cumulative number of Xpert MTB/RIF and LPA instruments per year, annual number of Xpert MTB/RIF assays, annual number of LPAs (stratified by first-line LPAs and second-line LPAs), laboratory-confirmed diagnoses using Xpert MTB/RIF and LPAs and drug susceptibility status for first-line drugs (rifampicin and isoniazid) and for second-line drugs (fluoroquinolones and aminoglycosides). For the fourth objective, data variables included the year, annual numbers of patients registered with TB, annual numbers of patients with laboratory-confirmed MDR/RR-TB and with XDR-TB and annual numbers of patients enrolled in MDR-TB and XDR-TB treatment. 

The source of the laboratory data was the electronic database in the Microsoft Excel^®^ spreadsheet at the National Reference Laboratory supplemented by paper-based laboratory record forms. The sources of clinical data were the regular routine reporting forms submitted by the regional centers of phthisiology and pulmonology to NTP headquarters. Data were collected between August and December 2020 into a Microsoft Excel^®^ (Microsoft Corporation, Albuquerque, NM, USA) spreadsheet.

### 2.5. Analysis and Statistics

A descriptive analysis was performed focusing on numbers, frequencies and proportions with comparisons made between the different years. Microsoft Excel^®^ for Office 365 Microsoft Online (16.0.12527.21490) 32-bit was used for collecting and handling data, as well as for performing the analysis and developing Tables and Figures.

## 3. Results

### 3.1. Numbers of GeneXpert and LPA Instruments Deployed Annually and Regionally 

**Figure 2:**
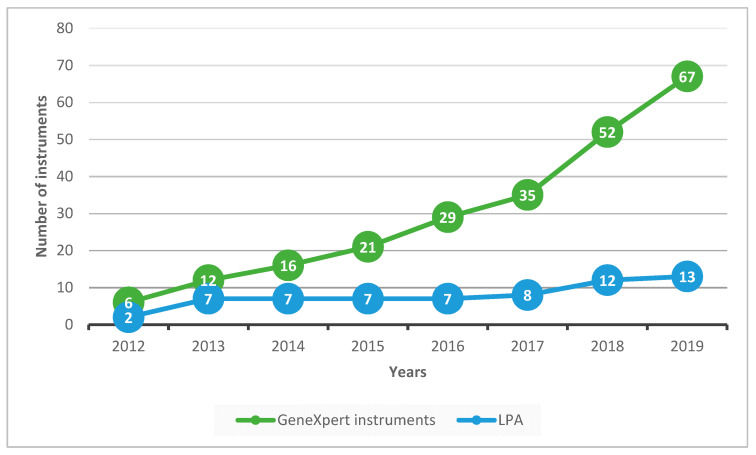
GeneXpert and LPA instruments cumulatively deployed in Uzbekistan from 2012 to 2019. LPA = line probe assay.

The numbers of GeneXpert and LPA instruments cumulatively deployed in the country between 2012 and 2019 are shown in Figure 2. GeneXpert instruments increased progressively from six to sixty-seven with the largest increases occurring between 2017 and 2019. LPA instruments increased more slowly from two to thirteen. 

The deployment of instruments in the regions and in relation to the regional population and regional TB incidence rates in 2019 are shown in Table 1. The majority of GeneXpert instruments were deployed in the Republic of Karakalpakstan followed by the Namangan region. Four instruments were placed in each of the five regions where TB notifications were >50 per 100,000. GeneXpert instruments were also deployed in the penitentiary sector, in HIV care and treatment centers and in two National Reference Laboratories. Per million population, GeneXpert deployment was highest in the Republic of Karakalpakstan followed by the Sirdaryo and Nawoiy regions. The Republic of Karakalpakstan and Tashkent city had the highest number of LPA instruments with the remainder of the regions having either one instrument or none. 

### 3.2. Numbers of Xpert MTB/RIF and LPA Diagnostic Tests Done Annually

**Figure 3:**
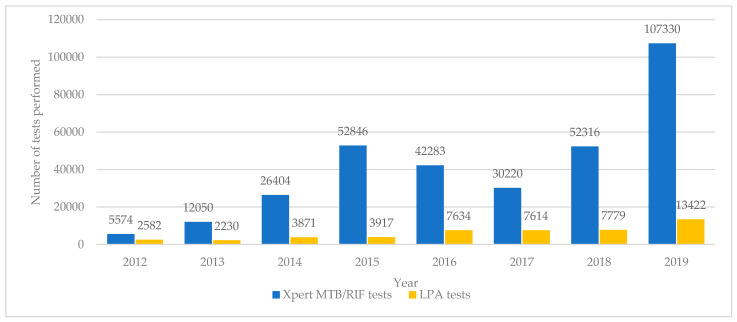
Molecular diagnostic tests (Xpert MTB/RIF and LPA) performed each year in Uzbekistan from 2012 to 2019. LPA = line probe assay.

The numbers of Xpert MTB/RIF and LPA tests done annually in Uzbekistan between 2012 and 2019 are shown in Figure 3. 

The number of Xpert MTB/RIF tests increased progressively each year from 5574 to 107,330 although in 2017 there was a decline in the number of tests performed. There were 2582 LPA tests carried out in 2012. These numbers stayed below 5000 until 2016 when there was an increase to above 7500 for the next three years and then a rapid jump to 13,422 in 2019.

**Figure 4:**
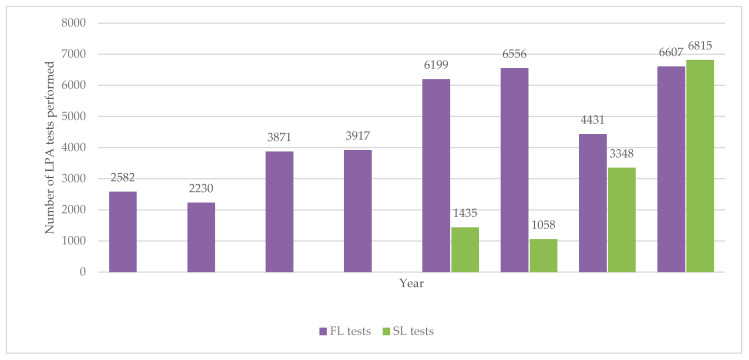
Number of LPA tests (first- and second-line) performed each year in Uzbekistan from 2012 to 2019. LPA = line probe assay; FL = first-line; SL = second-line.

The numbers of first-line and second-line LPA tests done annually between 2012 and 2019 are shown in Figure 4. The numbers of first-line LPA tests increased up to above 6000 by 2016 and then with the exception of one year remained at this level. Second-line LPA tests started in 2016 and the numbers rapidly increased in each of the following years. 

### 3.3. Performance and Results of Xpert MTB/RIF and LPA Tests

The performance and results of Xpert MTB/RIF tests from 2012 to 2019 are shown in Table 2. 

In the first five years, the proportion of successful tests varied from 90% to 94% but from 2017 onwards this increased to between 96% and 97% each year. In the first two years, about one third of successful tests detected *MTB* but this proportion declined in the following years to 11% in 2019. The proportion of detected *MTB* showing rifampicin-resistance varied throughout the period being at the highest in 2012 and 2018 and the lowest in 2019. Indeterminate results on rifampicin testing were generally below 5% except for 2015 when this figure was 6%. 

The performance and results of first-line LPA tests carried out between 2012 and 2019 are shown in Table 3. 

In 2013 and 2018 the proportion of tests showing rifampicin and isoniazid sensitivity/resistance did not add up to 100% of tests detecting *MTB* because a few of the tests showed indeterminate results.

The detection of *MTB* was ≥90% in the first two years but thereafter *MTB* detection was less than 90% and between 2015 and 2017 it was below 75%. The proportion of detected *MTB* showing rifampicin-resistance varied each year between 34% and 51% while isoniazid resistance varied between 28% and 72%. 

The performance and results of second-line LPA tests carried out between 2016 and 2019 are shown in Table 4. 

The detection of *MTB* decreased over the four years from 90% to 51%. The proportion of detected *MTB* showing fluoroquinolone resistance varied between 26% and 35% while that of aminoglycoside resistance varied between 5% and 35%. In 2019, over one third of tests for aminoglycoside resistance/sensitivity were not done.

### 3.4. Numbers with Laboratory-Confirmed MDR/RR-TB and XDR-TB and Numbers Enrolled on Treatment

The numbers and proportions of patients with laboratory-confirmed MDR/RR-TB and XDR-TB between 2012 and 2019 along with patients registered with all types of TB are shown in Table 5.

Annually, between 1728 and 2265 patients had laboratory-confirmed MDR/RR-TB (this constituted 10% to 13% of patients registered with all types of TB) with the exception of 2013 and 2014 where numbers were ≥5000 (constituting 20%–25% of all types of TB). The proportions of all TB patients with XDR-TB remained below 1% between 2012 and 2015 but thereafter the numbers increased to reach 602 (3.7% of all types of TB) in 2019. 

**Figure 5:**
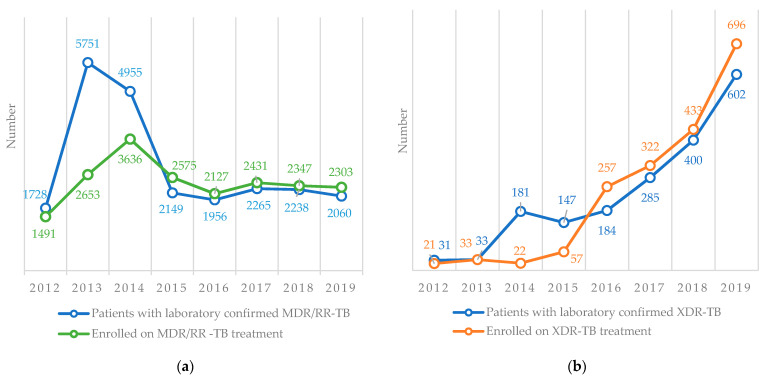
Number of patients with laboratory-confirmed MDR/RR-TB and XDR-TB and the number enrolled on treatment in Uzbekistan from 2012–2019. (**a**) MDR/RR-TB, (**b**) XDR-TB. TB = tuberculosis, MDR/RR-TB = multidrug-resistant/rifampicin-resistant TB, XDR-TB = extensively drug-resistant TB.

The numbers of patients with laboratory-confirmed MDR/RR-TB and XDR-TB and the numbers enrolled on treatment are shown in Figure 5. For MDR/RR-TB, there was a gap between the numbers confirmed and enrolled on treatment between 2012 and 2014 (the gaps were 14% in 2012, 54% in 2013 and 27% in 2014). Thereafter, there were more patients enrolled on the treatment than confirmed (Figure 5a). For XDR-TB, there was a similar pattern with a gap between the numbers confirmed and enrolled on treatment between 2012 and 2015. Thereafter, there were more patients enrolled on treatment than confirmed in the laboratory (Figure 5b).

## 4. Discussion

This is the first study from Uzbekistan documenting the national scale-up, use and results of molecular diagnostic tests between 2012 and 2019 along with numbers confirmed in the laboratory and numbers enrolled on treatment. There were a number of important findings. 

First, a good progress was made with the introduction and national scale-up of molecular diagnostic instruments that aligned with the recommendations from the WHO [4,6,7]. The number of GeneXpert instruments in the country progressively increased particularly in the last three years and, in general, deployment was in line with the regional population size and regional TB incidence rates. There was, however, a greater concentration of GeneXpert instruments in the Republic of Karakalpakstan where Médecins Sans Frontières (MSF) has been working since 2003 and supporting MDR-TB treatment optimization since 2013 [17,18,19]. GeneXpert instruments were also deployed in HIV care facilities to support the screening and detection of TB amongst people living with HIV who are at the highest risk of TB [20] and in the penitentiary sector where there is also a high burden of TB and DR-TB transmission [21]. Smaller numbers of LPA instruments were deployed in the country and all to the reference and regional laboratories. LPAs need to be placed in well-resourced higher-level laboratories that have trained and skilled laboratory technicians with optimal performance requiring meticulous attention to the manufacturer’s instructions about process, timing, sequencing and temperature control [5]. 

Second, the number of molecular diagnostic tests progressively increased in the country mirroring the increased number of deployed instruments. In 2019, there were 107,330 Xpert MTB/RIF tests performed using 67 instruments working out at an average of about 1500 tests per instrument. The decrease in numbers of tests performed in 2017 was due to stock outs of Xpert MTB/RIF cartridges, thus illustrating the importance of maintaining secure and reliable consumable pipelines. We did not assess the functionality of GeneXpert instruments but this is highly dependent on stable and regular electricity, adequate maintenance and basic computer training [22] and challenges with all of these issues can result in poor or interrupted usage of this diagnostic tool [23]. Poor utilization of instruments may also be a problem when countries start using the new technology for the first time and this may be particularly so in the more rural areas and in district hospitals [24,25,26]. At the start of scale-up, between 8% and 10% of Xpert MTB/RIF tests were unsuccessful but performance improved and in the last three years unsuccessful tests averaged just 3–4% each year. Similar levels of performance have been observed elsewhere [24,25,26]. 

The number of first-line LPA tests increased between 2012 and 2016 and thereafter remained fairly stable. A few second-line LPA tests had been performed prior to 2016, essentially in the Republic of Karakalpakstan under the support of MSF (these data were not available at the NTP and were not included in our study) but national scale-up really took place from 2016 onwards. In 2019, there were more second-line LPA tests conducted compared with first-line LPA tests. 

Third, in terms of *MTB* detection, about one third of Xpert MTB/RIF tests showed positive results in 2012 and 2013 with the proportion of *MTB* diagnoses gradually declining thereafter. These findings are probably due to changing international and national guidance about the use of Xpert MTB/RIF. When MTB/RIF was first deployed globally, the WHO recommended its use just for persons suspected of having MDR-TB and for those with presumptive TB who were also co-infected with HIV [27]. In 2013, the WHO recommended that Xpert MTB/RIF be considered as the initial diagnostic test for all people requiring an investigation for TB [4] and in 2017 advised that national programs transition away from smear microscopy and prioritize the initial use of Xpert MTB/RIF [2]. The broader use of Xpert MTB/RIF as a TB diagnostic tool is the likely reason for the decreasing proportion of tests diagnosing *MTB* and diagnosing rifampicin resistance. In 2017 the WHO recommended that Xpert MTB/RIF Ultra replace the first generation Xpert MTB/RIF [28]. This has higher sensitivity, is run on the same instrument and requires only a software upgrade and Uzbekistan is already phasing in this new software. 

The detection of *MTB* with first-line and second-line LPA tests depends on laboratory processes. If a pure culture of *MTB* is used for the assay then *MTB* case detection approaches 100%. However, if a sputum specimen is used either from a smear-positive patient or directly from a person with presumptive TB, then lower *MTB* yields will be obtained. A mixture of these different specimens was used over the eight years, resulting in variable yields of *MTB*. Unfortunately, we were unable to collect information about what specimens were used or how the tests were conducted. In terms of drug susceptibility, about half of the patients investigated by first-line LPAs had rifampicin or isoniazid resistance and about one quarter to one third of those investigated had fluoroquinolone resistance. There was a variable amount of aminoglycoside resistance with over one third of tests for aminoglycosides not being undertaken in 2019. The likely reason was the removal of aminoglycosides from the WHO priority group of drugs for drug-resistant TB because of toxicity issues [29]. 

Fourth, the scale-up and use of these molecular diagnostic tests led to a stable number of MDR-TB patients and an increasing number of XDR-TB patients being diagnosed each year. The large number of MDR-TB patients diagnosed in 2013 and 2014 probably reflected a large backlog from previous years where CDST was the principal and more complicated diagnostic method and the introduction of Xpert MTB/RIF assays resulted in better universal coverage and access. 

In the early years of this study, there was a significant gap between being diagnosed and starting on treatment for both MDR-TB and XDR-TB. We did not assess the reason for this but a lack of available drugs may have been an important factor. The gap between diagnosis and treatment is an issue not confined to Uzbekistan. Studies from Africa, Asia and the Western Pacific have shown that up to 40% of patients diagnosed with sputum smear-positive or culture-positive TB fail to start treatment [30] with results being no better with the use of Xpert MTB/RIF [31,32,33]. In the last four years, more patients were enrolled on treatment for MDR-TB and XDR-TB than confirmed in the laboratory. This may be a result of patients being diagnosed and treated on clinical grounds or because patients interrupted treatment due to adverse drug events and were enrolled again in the same year, resulting in double counting. As we used aggregate data, we cannot answer these questions and we also do not know whether all laboratory-confirmed patients did in fact get treated. 

The strengths of this study were the full national sample over a period of eight years and the conduct and reporting of the study in line with the Strengthening the Reporting of Observational Studies in Epidemiology (STROBE) Guidelines [34]. There were, however, a few limitations. The aggregate data were obtained from routine recording and reporting systems and there may have been errors that we were unable to identify. The use of aggregate data meant that we could not track whether individual laboratory-confirmed patients with MDR-TB and XDR-TB were enrolled on to treatment. Finally, we did not collect any data on laboratory procedures that would have helped gain a better understanding of some of the laboratory results. 

Despite these limitations there are three important programmatic implications. First, we need a better understanding of how GeneXpert instruments are used and their functionality (namely, are they in use all year round and are they being used to their full potential?). Studies on functionality issues in other countries have revealed that the instruments could be far better used [26]. There is a need to also understand how LPA tests are used. Consideration should be given to further decentralize the Xpert MTB/RIF assay by using new forthcoming technology; for example, Xpert OMNI, which is portable and battery-operated [35]. Second, there has been a huge increase in the use of second-line LPA tests and this has been mirrored by an increase in XDR-TB. The country needs to decide whether to procure more LPA instruments or move to a more rapid point-of-care GeneXpert platform (Xpert/XDR) that has been shown to accurately diagnose fluoroquinolone and other second-line drug resistance [36,37,38]. Improved and rapid diagnosis of second-line drug resistance will be key to the successful scale-up of an effective short course treatment for MDR-TB as recommended by the WHO [29] as well as a more effective treatment for XDR-TB using a combination of bedaquiline, pretomanid and linezolid (BPaL) [39]. Third, we need to conduct an individual cascade of care to determine whether patients with laboratory-confirmed MDR-TB and XDR-TB start treatment and in a timely way. A digital platform (GxAlert) is now available electronically and in real-time to connect GeneXpert instruments and assay results with clinics, doctors and patients [40,41]. This is already being piloted in Uzbekistan to see whether it improves linkage of diagnosis to care. 

## 5. Conclusions

The primary goal of the Uzbekistan NTP is to end the national TB epidemic by 2030 by reducing TB incidence and TB mortality. Drug-resistant TB poses an important threat to this achievement. Molecular technology provides a potential solution by allowing more rapid and accurate diagnosis and thereby more effective treatment. The current study showed that between 2012 and 2019 there has been good progress with the scale-up of molecular diagnostic instruments and assays in Uzbekistan. This has allowed better coverage and access for diagnosing rifampicin and isoniazid resistance as well as resistance to fluoroquinolones and aminoglycosides. The numbers of confirmed MDR-TB patients have remained fairly stable in the country over the last five years but there has been a large increase in the numbers of patients with laboratory-confirmed XDR-TB. Aggregate data suggest that most patients with laboratory-confirmed drug resistance are enrolled on to treatment. The study has allowed us to make several programmatic recommendations that we believe will further improve our efforts to control drug-resistant TB in the country. 

## Figures and Tables

**Table 1:** Number of GeneXpert and LPA instruments deployed in Uzbekistan in 2019 by region.

Region	Population, as of January 2020	TB Notifications per 100,000	Number of GeneXpert Instruments	GeneXpert Instruments per 1,000,000	Number of LPA Instruments
Republic of Karakalpakstan	1,916,400	90	8	4.2	2
Andijon region	3,172,300	45	4	1.3	0
Bukhara region	1,939,600	48	4	2.1	1
Jizzakh region	1,403,200	52	4	2.9	0
Qashqadaryo region	3,317,800	37	4	1.2	1
Nawoiy region	1,009,200	42	3	3.0	1
Namangan region	2,852,600	42	5	1.8	1
Samarqand region	3,928,600	57	4	1.0	1
Surkhondaryo region	2,667,100	36	3	1.1	1
Sirdaryo region	857,100	57	3	3.5	0
Tashkent region	2,978,100	51	4	1.3	1
Farghona region	3,803,000	39	4	1.1	1
Khorazm region	1,884,000	45	3	1.6	0
Tashkent city	2,654,100	40	4	1.5	3
**Uzbekistan**	**34,383,100**	**47**	67 *	**1.9**	**13**

* This includes three GeneXpert instruments placed in the penitentiary sector, five in HIV care facilities and two in National Reference Laboratories. TB = tuberculosis; LPA = line probe assay.

**Table 2:** Performance and results of Xpert MTB/RIF tests in Uzbekistan from 2012 to 2019.

Xpert MTB/RIF Tests	2012	2013	2014	2015	2016	2017	2018	2019
Tests performed, *n*	5574	12,050	26,404	52,846	42,283	30,220	52,316	107,330
Tests successfully completed, *n* (%)	5005 (90)	11,116 (92)	24,828 (94)	48,776 (92)	39,776 (94)	29,271 (97)	50,849 (97)	103,144 (96)
Tests detecting *MTB*, *n* (%) ^a^	1536 (31)	4032 (36)	6023 (24)	9214 (19)	7388 (19)	5122 (17)	9840 (19)	11,132 (11)
Rifampicin resistance, *n* (%) ^b^	631 (41)	n/a	2149 (36)	2765 (30)	2199 (30)	1666 (33)	4043 (41)	2664 (24)
Rifampicin sensitive, *n* (%) ^b^	880 (57)	n/a	3628 (60)	5894 (64)	4886 (66)	3397 (66)	5703 (58)	7998 (72)
Rifampicin indeterminate, *n* (%) ^b^	25 (2)	n/a	246 (4)	555 (6)	303 (4)	59 (1)	94 (1)	470 (4)

*MTB* = *mycobacterium tuberculosis*, n/a = data not available, ^a^ = % of successfully completed tests, ^b^ = % of tests detecting *MTB*.

**Table 3:** Performance and results of first-line LPAs in Uzbekistan from 2012 to 2019.

LPA FL Tests	2012	2013	2014	2015	2016	2017	2018	2019
Tests performed, *n*	2582	2230	3871	3917	6199	6556	4431	6607
Tests detecting *MTB*, *n* (%) ^a^	2314 (90)	2058 (92)	3444 (89)	2605 (67)	4406 (71)	4421 (67)	3452 (78)	5869 (89)
Rifampicin resistance, *n* (%) ^b^	1182 (51)	892 (43)	1460 (42)	1278 (49)	2078 (47)	1729 (39)	1427 (41)	1976 (34)
Rifampicin sensitive, *n* (%) ^b^	1132 (49)	1014 (49)	1984 (58)	1327 (51)	2328 (53)	2692 (61)	1902 (55)	3893 (66)
Isoniazid resistance, *n* (%) ^b^	1425 (62)	1091 (53)	1736 (50)	1080 (41)	2354 (53)	1230 (28)	1658 (48)	2530 (43)
Isoniazid sensitive, *n* (%) ^b^	889 (38)	814 (40)	1708 (50)	1525 (59)	2052 (47)	3191 (72)	1671 (48)	3339 (57)

LPA = line probe assay, FL = first-line, *MTB* = *mycobacterium tuberculosis*, ^a^ = % of tests performed, ^b^ = % of tests detecting *MTB*.

**Table 4:** Performance and results of second-line LPAs in Uzbekistan from 2012 to 2019.

LPA SL Tests	2016	2017	2018	2019
Tests performed, *n*	1435	1058	3348	6815
Tests detecting *MTB*, *n* (%) ^a^	1298 (90)	789 (75)	1693 (51)	3482 (51)
FQ resistance, *n* (%) ^b^	336 (26)	157 (20)	587 (35)	1148 (33)
FQ sensitive, *n* (%) ^b^	962 (74)	632 (80)	1106 (65)	2334 (67)
Aminoglycoside resistance, *n* (%) ^b^	64 (5)	15 (2)	598 (35)	376 (11)
Aminoglycoside sensitive, *n* (%) ^b^	1234 (95)	774 (98)	1095 (65)	1776 (51)

LPA = line probe assay, SL = second-line, *MTB* = *mycobacterium tuberculosis*, FQ = fluoroquinolones, ^a^ = % of tests performed, ^b^ = % of tests detecting *MTB*. In 2019, 38% of tests for aminoglycoside sensitivity/resistance were not done. The most probable reason was the removal of aminoglycosides from a priority grouping of drugs for the treatment of drug-resistant tuberculosis by the WHO Consolidated Guidelines Development Group in 2018.

**Table 5:** Number of patients with all types of TB and the number with laboratory-confirmed MDR/RR-TB and XDR-TB in Uzbekistan from 2012 to 2019.

Patients with TB	2012	2013	2014	2015	2016	2017	2018	2019
All types of TB, *n*	16,810	25,168	22,804	19,055	18,441	19,329	18,496	16,272
MDR/RR-TB, *n* (%) *	1728 (10.3)	5751 (22.9)	4955 (21.7)	2149 (11.3)	1956 (10.6)	2265 (11.7)	2238 (12.1)	2060 (12.7)
XDR-TB, *n* (%) *	31 (0.2)	33 (0.1)	181 (0.8)	147 (0.8)	184 (1.0)	285 (1.5)	400 (2.2)	602 (3.7)

TB = tuberculosis, MDR/RR-TB = multidrug-resistant/rifampicin-resistant TB, XDR-TB = extensively drug-resistant TB, * = % of all types of TB.

## Data Availability

The data that support the findings of this study are available from the corresponding author, S.Y., upon reasonable request.
